# The bidirectional effects of obsessive-compulsive symptoms and difficulties in emotion regulation in Chinese adults during the COVID-19 pandemic—a dynamic structural equation model

**DOI:** 10.1186/s40359-022-00841-5

**Published:** 2022-05-21

**Authors:** Danping Hong, Yawen Zhu, Runting Chen, Bihong Xiao, Yueyi Huang, Meng Yu

**Affiliations:** 1 Guangdong Provincial Key Laboratory of Social Cognitive Neuroscience and Mental Health, Department of Psychology, Sun Yat-Sen University, Guangzhou, 510006 People’s Republic of China; 2 Key Laboratory of Behavioral and Mental Health of Gansu Province, School of Psychology, Northwest Normal University, Lanzhou, 730070 People’s Republic of China; 3 School of Psychology, South China Normal University, Guangzhou, 510631 People’s Republic of China

**Keywords:** COVID-19, Difficulties in emotion regulation, Dynamic structural equation modeling, Obsessive–compulsive symptoms, Sleep problems

## Abstract

**Background:**

With the accumulation of negative emotions brought by COVID-19-related dysfunctional beliefs, individuals adopted obsessive–compulsive (OC) symptoms (e.g., over-checking the wearing of masks) and formed difficulties in emotion regulation (DER). This study focused on the temporal dynamics of the bidirectional relation between OC symptoms and DER, which had a devastating effect on the individual's mental health. As an extension, we further explored whether OC and DER and their relationship affect sleep problems.

**Methods:**

In February 2020, a 14-day (twice a day, of 28 measurement intervals) online questionnaire survey was conducted on 122 Chinese adults (aged 18–55 years; 63 females). Subsequently, this research applied a dynamic structural equation model with a cross-lagged relationship and a time series. Health anxiety, anxiety, and depression were controlled as covariates.

**Results:**

Both OC symptoms and DER had a significant autoregressive and cross-lagged effect. Comparatively speaking, DER was a stronger predictor of OC symptoms than OC’s prediction of DER. Moreover, both higher levels of OC symptoms and DER were related to the severity of sleep problems.

**Conclusions:**

More guidance on intervening in OC symptoms and identifying emotion regulation should be added to reduce the negative impact of the COVID-19 pandemic on public mental health.

**Supplementary Information:**

The online version contains supplementary material available at 10.1186/s40359-022-00841-5.

## Introduction

With the massive outbreak of COVID-19 at the end of January 2020, the Chinese government called the populace at home and not necessarily go outside. The public needed to wear masks, detect body temperature, and maintain social distance when they got in and out of the streets, public transport, and other places, which had become the social norms to counter the threat of virus transmission.

Obsessive–Compulsive (OC) symptoms are composed of intrusive thoughts and repetitive behaviors, essentially suppressing or preventing excessive emotional expression [1, 2]. OC symptoms in COVID-19-related social norms refer to over-checking, over-washing, obsessing, and metal neutralizing. Several kinds of dysfunctional beliefs might induce negative emotions and evaluations, for instance, exaggerating external threats, paying too much attention to the correctness of their thoughts, and trying to control them, not tolerating uncertainty and perfectionism. During the epidemic, when intrusive thoughts (e.g., exaggerating the possibility of infection with a virus) came, compulsive behaviors would be used to alleviate the pain of negative emotions temporarily. However, the repeated execution of the compulsions can also accumulate painful emotions in the long term, especially when people lack an understanding of emotions and confidence in regulating painful emotions [3, 4]. Due to the existence of these negative beliefs, people would continue compulsive behaviors, resulting in a vicious circle. Repeated OC symptoms will solidify OC disorders [5].

Difficulties in emotion regulation (DER) showed that individuals respond to their negative emotions in an unbalanced way [6]. Gratz and Roemer identified four aspects involved in emotion dysregulation: (1) poor awareness and understanding of emotions, (2) poor acceptance of emotions, (3) lack of the ability to engage in goal-directed behaviors and refrain from impulsive behaviors when experiencing negative emotions, and (4) access to maladaptive emotion regulation strategies. Previous studies indicated that many psychological symptoms based on avoiding internal experiences are directly related to DER [7]. In the face of crises and a forced social quarantine, the public needed to adjust their negative emotions promptly. However, individuals with DER are more challenging to adapt to life under the COVID-19 epidemic and easily suffer from psychological problems [8].

Gross [9] proposed that the process of emotion regulation is shown as “situation selection → allocation of attention → appraisal → response.” Calkins et al. [3] expanded Gross’s model and considered that compulsions are a maladaptive emotion regulation strategy. It takes suppression during the response process, in which people try to reduce emotional expression. Moreover, plenty of cross-sectional studies has confirmed the intimate relationship between OC and DER. For instance, Stern et al. [5] conducted a study on undergraduates (*n* = 170) and the results showed that OC symptoms were significantly correlated with poor understanding and fear of negative and positive emotions. Fergus and Bardeen [10] showed that difficulties in impulse control and lack of emotional clarity were uniquely associated with each dimension of OC symptoms. In addition, Yap et al. [11] suggested that non-acceptance of emotions and non-participation in goal-oriented behaviors were markedly associated with OC across samples. In clinical samples (*n* = 59) and non-clinical samples (*n* = 331), even if anxiety, depression, and demographic variables were controlled, the positive correlation between OC and DER was still established [11]. Although some cross-sectional studies obtained a significant mediation path that DER affected OC, this inference was not causally persuasive. For instance, Eichholz et al. [12] revealed that DER played a mediating role in self-compassion affecting OC symptom severity in patients (*n* = 90).

Combined with previous research and the epidemic's situation, it can be inferred that OC symptoms and DER are more likely to present a mutual influence. Individuals lacking effective and adaptive emotion regulation strategies during the epidemic would rely on current social norms to alleviate negative emotions. Influenced by dysfunctional beliefs, intrusive thoughts would make individuals more inclined to adopt maladaptive compulsions to avoid, un-clarify, uncomprehending, and un-accept their negative and distressing emotions, which further promote the formation of DER. Besides, if the vicious circle begins, it makes them less confident in regulating emotions and difficult to control their impulsive behaviors. To conclude, discussing the relationship between OC and DER under the epidemic environment would guide the public in proper response strategies when facing an emergency public crisis.

Following the predecessors' suggestion, when discussing the relationship between OC and DER, the effects of anxiety and depression should be considered as covariates. Previous research has indicated that anxiety and depression were closely related to OC symptoms and DER [11]. When confronted with the health threats brought by COVID-19, individuals are prone to worry about their and their close families and friends’ health, which quickly leads to health anxiety [13]. Health anxiety (HA) refers to the state that individuals overly worry about getting sick, exaggerate their physical feelings, and negatively explain physical symptoms [14]. According to the cognitive model of HA [14], it will further produce distorted beliefs, destructive emotions, and maladaptive behaviors, resulting in more OC symptoms and DER [15, 16]. During the COVID-19 lockdown, individuals barely went to the hospital for a diagnosis in time or mainly relied on work to be distracted. In that case, they were supposed to be more likely to use compulsions to get rid of emotional distress. Therefore, HA may also play an essential role in the relationship between the association of OC symptoms and DER.

Sleep problem is also an important issue that cannot be ignored during social isolation [17]. As an extension, we explored the predictive effect of the relationship between OC symptoms and DER on daily sleep. Sleep problems (SP), including hard to fall asleep, waking up early, and not getting enough sleep, were the COVID-19-related features, which are also part of the characteristics of sleep disorders in DSM-5 [2]. Past research has found intrusive thoughts, uncontrollable worries, and other cognitive arousals that may hinder sleep onset and cause insomnia [18]. Riemann et al. [19] proposed a hyperarousal model of insomnia. Concretely, the model pointed out that psychological stress before going to bed (e.g., intrusive thoughts) and the dysregulation of emotion regulation are accompanied by excessive reflection, which leads to a variety of SP. In a cross-sectional study with non-clinical samples, similar results were obtained, especially that obsessions aggravated insomnia [20]. Compulsions also predict sleep time reduction and sleep loss [21]. Moreover, SP was considered to be related to the accumulation of negative emotions, decreased positive emotions, and insufficient emotional regulation [22]. A longitudinal study of three years (*n* = 942) has examined that DER positively predicted SP, and DER played an intermediary role in social relationships and SP [23]. Moreover, a review of 44 cross-sectional epidemic articles worldwide summarized multiple factors that could lead to SP [24], such as negative emotions, stress, and the deficiency of social support. Nevertheless, no research has discussed OC symptoms in cross-sectional studies and used longitudinal analysis during the COVID-19 pandemic. It can be inferred that both OC and DER could affect SP, and even OC would further impact SP through DER.

So far, only a few studies have combined longitudinal research design to measure the same batch of subjects to psychological changes of OC symptoms or DER. For example, a study in the UK (*n* = 1958) used four-time points to indicate the association between loneliness and depressive symptoms, finding that DER was not the moderator of temporal interaction [8]. These conventional longitudinal studies collected developmental process data concerning between-person change and covariates affecting change. However, the ambulatory assessment highlights its advantage in terms of timeliness and acuity recording the subtle changes in the public's psychological states [25]. The stable process data measured by ambulatory assessment would fluctuate around the mean, focusing on within-person variability and covariates predicting when values deviate from the mean [26]. It provides daily measurement to draw a more stable causal inference for OC and DER.

To conclude, the present study used an ambulatory assessment design with a sample of 122 Chinese adults to conduct questionnaires twice in the morning and evening separately for 14 consecutive days, with 28 measurement intervals in total. The Dynamic Structural Equation Modeling (DSEM) [27] framework was utilized to create a multilevel cross-lagged model that examined the bidirectional relationships between OC symptoms and DER. Next, to ensure the reliability of causal inferences, we also added factors related to the two variables and the epidemic—the level of health anxiety, anxiety, and depression—as covariates. As an extension, we further tested the predictive effect of the two-way relationship on SP.

## Method

### Participants

Of the 122 subjects included in the final sample, their ages ranged from 18 to 55 (*M* = 22.18, *SD* = 7.03), with 69 females (56.6%). Most of the participants (83.6%) have a bachelor's degree or above. The sample consisted of participants coming from 18 provinces in 50 cities in China. There were 12 participants (9.8%) coming from severe epidemic areas and 25 (20.5%) from extremely severe epidemic areas (see Additional file 1: Table S1 for more information).

### Procedures

The present study was approved by the corresponding author’s affiliation ethic review board. Questionnaires were issued by the Wenjuanxing website (https://www.wjx.cn/). First, we collected baseline data on mental health variables from the Chinese adults from February 1, 2020. Next, participants volunteered to join our online WeChat group for follow-up research. Beginning on February 2, at 9 am and 9 pm for 14 consecutive days, the research assistant sent out a questionnaire link to the WeChat group. Subsequently, participants logged into the questionnaire with the same experiment number and needed to complete it within 2–3 min.

### Measures

#### Daily measures

Obsessive–Compulsive Symptoms. The study chose the four most relevant items to the pandemic situation from Obsessive–Compulsive Inventory-Revised (OCI-R) [1, 28], including washing, checking, obsessing, and mental neutralizing. The scale is a five-point score from 1 (*not at all*) to 5 (*extremely*). In the present study, the intraclass correlation coefficient (ICC) [29] was adopted to represent the within-person differences accounted for the proportion of the total variance, which was often used to assess the fit of stratified data in ambulatory assessment research. The ICC for OC symptoms was 0.90.

Difficulties in Emotion Regulation. The study used the Chinese version of the Difficulties in Emotion Regulation Scale (DERS) [6, 30], from which one item in each dimension, six items in total, was selected. The six items included difficulties in engaging in directed behaviors, limited access to emotional regulation strategies, nonacceptance of emotional responses, difficulties in controlling impulsive behaviors under negative emotions, lack of emotional clarity, and lack of emotional awareness. The COVID-19-related adapted version was in the premise of "*When I feel pressure due to the epidemic*." It is a 5-point Likert scale, with a score ranging from 1 (*totally disagree*) to 5 (*totally agree*). The ICC for DER was 0.85.

Sleep Problems. The assessment combined the Insomnia Severity Index (ISI) [31] and the PROMIS Sleep Disturbance—Short Form^1^ [32]. Three items were selected: (1) difficulties with falling asleep; (2) waking up too early; (3) difficulties with staying asleep. The score ranged from 1 (*completely inconsistent*) to 5 (*very consistent*), and it was only measured once a day in the morning. The ICC for SP was 0.75. The higher the score of the items, the more serious the sleep problems. According to the suggestion by Asparouhov et al. [27], SP's value was proportionally processed as a categorical variable in the between-level. Specifically, the severity of daily sleep problems accounting for 14 days was divided into mild, moderate, and severe.

#### Baseline measures

*Health anxiety* The Chinese version of the Short Health Anxiety Inventory contains 18 items with good reliability and validity (SHAI) [34, 35]. And it consists of 4 statements ranging from 0 (*I spend very little time)* to 3 (*I spend most of my time*). The Cronbach’s alpha was 0.88.

*Anxiety* The Self-Rating Anxiety Scale (SAS) is widely used and has good reliability and validity [36, 37]. It contains 20 questions, from 1 (*I spend very little time*) to 4 (*I spend most of my time*) to score. The Cronbach’s alpha in the current study was 0.73.

*Depression* The PROMIS Emotional Distress—Depression—Short Form was used to measure participants’ depressive symptoms^2^ [38]. The CFA model fitting indexes of this scale can be verified in another cross-sectional sample (*n* = 1,733) collected by our team during the same period, i.e., *x*^2^/*df* = 4.782, CFI = 0.995, TLI = 0.991, RMSEA = 0.037, SRMR = 0.011, showing a well-fit model [39]. The scale contains eight questions, scoring from 1 (*never*) to 5 (*almost always*). The Cronbach’s alpha of the present study was 0.81.

### Data analyses

First of all, IBM SPSS Version 25 conducted descriptive and correlational analyses. Then, we built a dynamic structural equation model using Mplus 8.3. Based on the Bayes estimator, DSEM is a simulation method with posterior distribution, combining the advantages of structural equation modeling, multilevel modeling, autoregressive cross-lagged modeling, and time-series modeling [27]. DSEM is an effective statistical method for analyzing intensive longitudinal data [40], allowing concurrently operating bivariate models. It has good applicability for small sample models [41]. Specifically, 50,000 Markov Chain Monte Carlo iterations and two chains were used to estimate parameters and handle missing data [42]. To be specific, the current study contained a total of 122 samples (*N*) and 28-time observation points (*T*, every 12-h), which was considered to have good statistical reliability by simulation research [43]. Research-designedly, each participant had 28 measurement points. Excluding the number of omissions, 3,368 data points were left, and the effective response rate was 99.1%.

The data analyses section included a two-level structure: repeated daily assessment data (within-person level) nested in an individual (between-person level). The within-person level calculated the relatively stable individual differences in correlation, autoregressive, and cross-lagged relationships [44]. Brose et al. [45] proposed that the two-level differences affecting structure were not equivalent but related. The within-person variability received attention when interpreting individual relevant emotional variables while the between-person differences provided unique insights into the internal function [45]. According to Schuurman et al. [41] suggestion, autoregressive and cross-lagged parameters in the within-person level should be standardized in comparison, while using unstandardized results in the between-person level. The cross-lagged parameter reflected the predictive relationship and represented a causal mechanism. However, making causal inferences needs to be aware that variables may simultaneously affect the two outcome variables to change the specific relationship [44]. It mainly detected means (fix effects), variances (random effects), and covariates at the between-level. The random effects were adopted to capture each person's deviation from the average trajectory [44]. If 95%CI did not contain 0, the path was significant.

In the first step, we calculated the bivariate relationship between OC symptoms and DER. The second step was to extend the model to predict SP by calculating OC symptoms, DER, and their bidirectional relationships. Finally, three baseline variables (health anxiety, depression, anxiety) were controlled as covariates. The M*plus* syntaxes are presented in the additional files.

## Results

### Descriptive statistics

OC symptoms and DER included 28 measurement points, and sleep problems contained 14 daytime points. The three variables (health anxiety, depression, anxiety) measured one point in baseline due to not changing over time and being relatively stable within the individuals. The results are shown in Table 1.

**Table 1:** The scales score on six variables among Chinese adults (*n* = 122)

Scales	Range	Means	SD
1.OCI-R	4–20	11.61	3.62
2.DERS	6–28	10.35	3.81
3.ISI	3–14	5.84	2.56
4.SHAI	19–49	29.94	7.31
5.SAS	20–59	31.41	7.63
6.PROMIS—depression	8–40	15.18	6.43

The scales in the first column successively correspond to six variables: obsessive–compulsive symptoms, difficulties in emotion regulation, sleep problems, health anxiety, anxiety, and depression

### Path correlation coefficients

The standardized regression correlation coefficients differed between the levels. At the within-level, the correlation between OC and DEP was 0.03 with 95%CI [0.00, 0.07]. At the between-level, after controlling for the covariates, the correlations between DER and P1 was − 0.37 with 95%CI [− 0.56, − 0.13]. And the correlation coefficient between P1 and P4 was − 0.26 with 95%CI [− 0.46, − 0.04]. The rest of the correlations showed no significance, which ranged from − 0.11 to 0.22.

### Within-person level model estimates

#### Autoregression effects

Autoregressive parameters, also known as inertia or carryover, indicate how quickly an individual can regain balance after being disturbed [44]. The standardized results showed that P1(OC_t−1_ → OC), i.e., the autoregressive value of OC, was positive (*β* = 0.48, 95%CI [0.44, 0.53]). It demonstrated that with severer OC symptoms in a period (of 12-h), it is more difficult for individuals to restore to their original level during the next period. The influence of P2(DER_t−1_ → DER), i.e., the autoregressive value of DER, was also positive (*β* = 0.35, 95%CI [0.30, 0.40]), which showed high inertia of DER the last time, and it would take a longer time to return to the baseline level in the following period. The autoregressive prediction effect of OC symptoms was significantly stronger than that of DER (0.48 vs. 0.35).

#### Cross-lagged effects

**Fig. 1:**
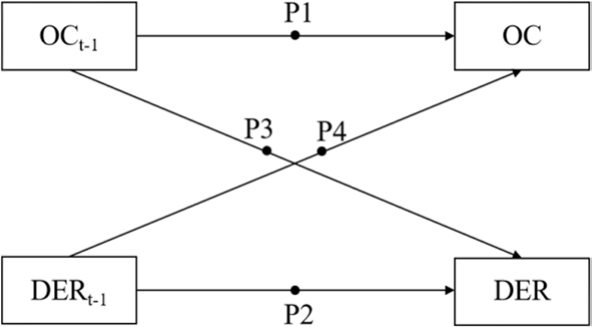
Autoregressive and cross-lagged parameter paths of the within-level. *Note* OC is obsessive–compulsive symptoms, and DER is difficulties in emotion regulation. P1 and P2 represent the autoregressive path of OC and DER, respectively, while P3 and P4 represent the cross-lagged paths. After controlling for the covariates, the effects were still significant

The cross-lagged parameters represent the cascade effect of functioning or behavior cross-domain and essentially reflect the predictive relationships [44]. On average, as shown in Fig. 1, P3(OC_t−1_ → DER), i.e., the cross-lagged parameter of OC affecting DER, was shown to be statistically significant and positive (*β* = 0.07, 95%CI [0.02,0.12]). And P4(DER_t−1_ → OC), i.e., the cross-lagged parameter of DER affecting OC, was also significant (*β* = 0.09, 95%CI [0.05,0.13]). Hence, it explained to a certain extent that both OC symptoms and DER would predict each other during the COVID-19 pandemic.

### Between-person level model estimates

#### Sleep problems

**Fig. 2:**
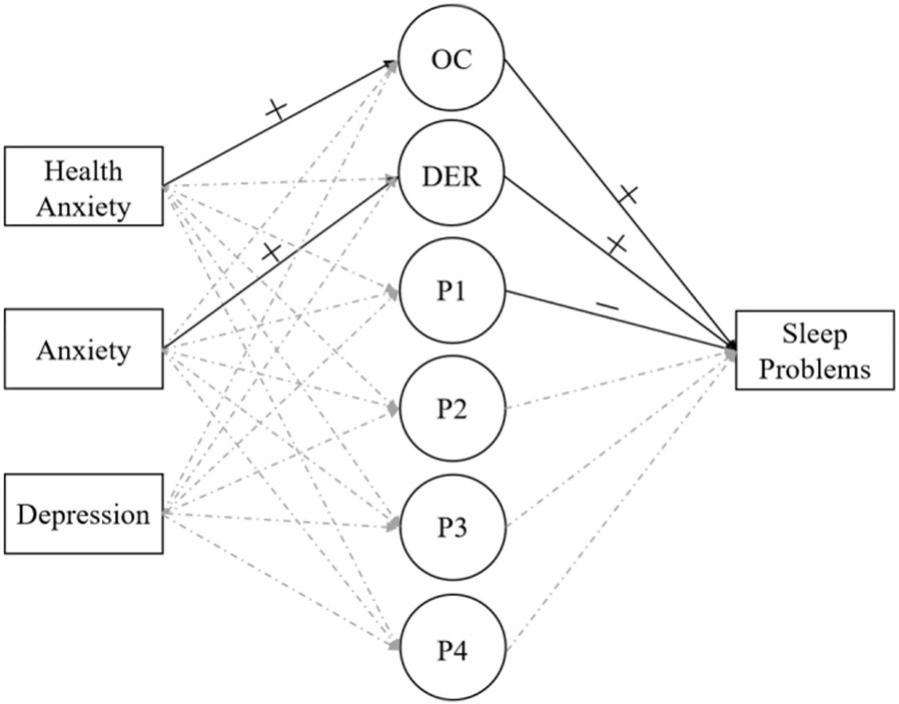
The between-level of Step 2. *Note* OC is obsessive–compulsive symptoms, while DER is difficulties in emotion regulation. P1, P2, P3, P4 are the autoregressive and cross-lagged parameters of OC and DER, respectively. At the between-level, time-invariant covariates were controlled for OC, DER, P1, P2, P3, and P4

According to the data analyses section, Table 2 and Fig. 2 show the results corresponding to the second step. Both OC and DER positively predicted SP, while P1 negatively predicted SP. To be specific, the slope of OC on SP was 1.07 with the 95%CI [0.05, 1.97]; DER affecting SP was 1.20 with the 95%CI [0.24, 2.20]; P1(OC_t−1_ → OC) negatively predicting SP was − 4.25, with 95%CI [− 7.43, − 0.95].

**Table 2:** Parameter estimates and 95% confidence interval of the between-level (*n* = 122)

Parameter	OC	DER	P1(OC_t−1_ → OC)	P2(DER_t−1_ → DER)	P3(OC_t−1_ → DER)	P4(DER_t−1_ → OC)
Step1						
Means	.05[− .64,.72]	.01[− .70,.70]	.51[.43,.61]*	.35[.28,.43]*	.09[.00,.17]	.09[.02,.17]*
Variances	10.79[7.69,15.27]*	12.63[9.16,17.87]*	.13[.09,.18]*	.07[.04,.11]*	.10[.05,.18]*	.12[.08,.18]*
Step 2						
Intercepts	− .02[− .59,.55]	− .04[− .51,.44]	.51[.42,.59]*	.35[.28,.43]*	.08[− .01,.17]	.09[.02,.17]*
Residual variances	6.73[4.78,9.44]*	5.40[3.98,7.54]*	.12[.09,.17]*	.07[.05,.10]*	.10[.06,.16]*	.11[.07,.16]*
Sleep problems	1.07[.05,1.97]*	1.20[.24,2.20]*	− 4.25[− 7.43,− .95]*	− 1.30[− 5.08,2.59]	− .48[− 4.54,3.62]	− .48[− 4.28,3.36]
Cov. (health anxiety)	.24[.15,.34]*	− .04[− .11,.04]	.01[− .01,.02]	.01[− .01,.02]	− .01[− .02,.01]	.01[− .01,.02]
Cov. (depression)	.01[− .10,.12]	.09[− .01,.19]	− .01[− .02,.01]	.01[− .00,.02]	− .01[− .02,.01]	.00[− .01,.02]
Cov. (anxiety)	− .04[− .14,.06]	.28[.19,.36]*	− .01[− .02,.01]	− .01[− .02,.01]	.01[− .01,.02]	− .01[− .02,.01]

Results are presented as unstandardized coefficients. *p < 0.05

#### Covariates

Health anxiety, anxiety, and depression were collected at the baseline measurement and were time-invariant variables. As viewed in Fig. 2, HA is a significant predictor of OC symptoms (*β* = 0.24, 95%CI [0.15, 0.34]). And anxiety positively predicted DER (*β* = 0.28, 95%CI [0.19, 0.36]). For every unit increase in HA, the level of OC symptoms would increase by 0.24 points, while for every unit increase in anxiety, the average level of people's DER would increase by 0.28 points. Moreover, for the means (fix effects) in all steps, only P1, P2, and P3 were significant, and the variances (random effects) of all variables were shown to be significant. The variances of OC and DER decreased after controlling for the covariates.

## Discussion and conclusions

In our 14-day ambulatory assessment study, a two-level DSEM was used to check the bidirectional relationship between OC symptoms and DER. First, the cross-lagged effects were tested in the OC and DER (step1). Next, the predictive effects of OC symptoms, DER, and their relationship, separately, on daily sleep problems were analyzed (step2). In addition, covariates were controlled to examine whether this relationship would change or still retain.

Results showed that both OC symptoms and DER had a significant autoregressive effect. After presenting OC and DER manifestations, participants needed to take a long time to recover to the baseline level in the next period (of 12-h), and OC was slower than DER to return to the baseline. And it indicated that individuals tended to repeatedly choose a period of high level of OC and DER or low OC and DER. This finding suggested that OC and DER have a long-term negative impact on the vicious circle.

The most important finding was that both OC symptoms and DER could predict each other. Results showed that, at the within-level, OC symptoms and DER were positively related, and the cross-lagged parameters of OC and DER were significant. Specifically, by contrast, DER was shown to be a larger predictor of OC symptoms. Similar findings have been found in previous cross-sectional studies, for instance, all dimensions of DER separately had a significant correlation with OC [10, 11]. Moreover, negative emotional reactions and poor adaptation to emotions were significantly and negatively related to the improvement of OC behaviors [46]. The higher the tendency of emotional regulation difficulties, the more difficult it was for individuals to recognize, accept and understand their own emotions [6]. In view of Gross [9, 47], taking OC behaviors is a means of suppressing or avoiding emotions. Therefore, when perceiving their anxiety, individuals are more likely to rely on repetitive and safe behaviors to eliminate emotional distress, i.e., individuals would excessively adopt OC to cope with the pandemic. In general, the vicious circle increased the DER level in the long run and increased the impact of DER on OC.

Furthermore, results showed that, as a COVID-19-related maladaptive emotion regulation strategy, OC symptoms could positively predict and maintain DER in a period. While this cross-lagged effect was smaller than the influence of DER on OC. This finding is consistent with the cognitive model of OC disorders [3]. Given the current pandemic situation, exaggerating that getting infections may be dysfunctional would push individuals to rely on social norms. Moreover, they adopted the excessive and repetitive implementation of social norms, which would be strengthened compulsions. These compulsions may include over-checking the wearing of masks and over-focusing on epidemic information. Intrusive and threatening thoughts that continue to break in are accompanied by strong negative emotions, especially anxiety, which could cause emotional pain [3, 5]. As a maladaptive emotion regulation strategy, compulsions only could temporarily relieve emotional distress [3]. However, frequent use of compulsions would accumulate affective distress, making individuals lose confidence in their ability to regulate their emotions, thereby deepening the tendency to avoid emotions [10]. When intrusive thoughts reappeared due to the lack of adaptive coping strategies, the vicious circle could only be repeated continuously. This vicious circle might destructively damage individuals' mental health by persisting in doing so.

In addition, the variances (random effect) of both OC and DER were decreased but still significant after controlling the covariates at the between-level. Especially, anxious emotions (including health anxiety and anxiety) showed a significant influence. In the COVID-19 pandemic, the higher the level of health anxiety, the higher the OC was, which was in line with the theory indicating that excessive worry about their health is a distorted cognition and will produce intrusive thoughts of illness [14]. In the present study, albeit as a covariate, depression was not shown to be a significant effect, which was inconsistent with Yap et al. [11]. A possible explanation was that, during the early COVID-19 epidemic, anxious emotion dominated other than depression due to the uncertainty about the future [48].

Thirdly, longitudinally, maintaining a higher level of OC and DER would trigger severer sleep problems. Combining with findings from Harvey [18] and Riemann et al. [19], they explained that, as the obsessions and compulsions brought about by intrusive thoughts before falling asleep, the increase in negative emotions and the decrease in positive emotions could affect the sleep process. In addition, it’s worth noting that the continuous maintenance of OC would relieve sleep problems. A possible fact was that the continuous appearance of OC symptoms (i.e., washing, checking, obsessing, and mental neutralizing) could instead assure individuals when facing potential threatening stimuli during a relatively short period (e.g., 12 h). This assurance would further lessen sleep problems.

The present research supports the importance of identifying and intervening OC symptoms and DER and their combined negative impacts on sleep problems. Specifically, the public should be wary that the repeated execution of behaviors in response to the epidemic is only a temporary emotional regulation. It is necessary to identify distorted beliefs and strengthen a correct understanding of the influence of the epidemic. Cisler and Olatunji [49] pointed out that improving the ability to regulate emotions could affect the negative emotional state caused by intrusive thoughts and improve the OC. Moreover, reasonable and adaptive emotional regulation strategies to guide were considered an effective response to eliminating the vicious cycle of OC symptoms [10]. Simultaneously, adaptive emotional strategies can effectively relieve insomnia [50]. Cognitive-behavioral therapy has proven to be effective in targeting both OC and DER [51, 52]. It carries out cognitive reconstruction and behavioral intervention and improves adaptive behaviors. Furthermore, cognitive-behavioral therapy intends to encourage the public to perceive and accept their own emotions and to learn more adaptive emotion regulation strategies, reducing the vicious cycles.

There are some limitations that should be mentioned. First of all, most of our measurement tools have been adapted based on the epidemic situation. Even if the reliability was acceptable or good, it weakened the extent of extension to other measurement occasions. Nevertheless, in the early stage of the epidemic, no epidemic-specific scales were developed to promptly record the dynamic changes in individuals’ mental states. Therefore, the advantages of such adaptation still outweighed the disadvantages. Second, most participants were college students, lacking special groups, such as clinical or sub-clinical participants. Then, we did not analyze the specific-person patterns, which may be able to find specific coping patterns and obtain valuable experience at the within-person level. Further research can use reliable tools, different sample groups, sample numbers, and time intervals to capture more residuals to support the reliability.

In conclusion, despite the limitations mentioned above, the present study was still the first to utilize DSEM to assess the bidirectional relationship between OC and DER and their association with sleep problems.

## Supplementary Information

**Additional file 1.** **Supplementary Table 1.** Sample Characteristics (*n* = 122). Supplementary Codes in Step1. Supplementary Codes in Step2.

## Data Availability

Materials described in the manuscript will be freely available to any researcher wishing to use them for non-commercial purposes when requesting for first or corresponding author.

## Abbreviations

COVID-19: Coronavirus disease of 2019
OC: Obsessive–compulsive
DER: Difficulties in emotion regulation
HA: Health anxiety
SP: Sleep problems
DSEM: Dynamic structural equation modeling
OCI-R: Obsessive–compulsive inventory-revised
ICC: Intraclass correlation
DERS: Difficulties in Emotion Regulation Scale
ISI: Insomnia Severity Index
SHAI: Short health anxiety inventory
SAS: Self-Rating Anxiety Scale

## Footnotes

1 The DSM-5 online scales were translated and been authorized by APA to the Chinese scholars in Beijing Normal University and Peking University of China to translate culturally. The original version has been proven to be psychometrically good [32, 33]. However, so far, it has not been culturally revised in any Chinese sample. Hence, in the present study, we provided confirmatory factor analysis results with another adult sample.

2 See Footnote 1.
