# Supplementary material for: The bidirectional effects of obsessive-compulsive symptoms and difficulties in emotion regulation in Chinese adults during the COVID-19 pandemic—a dynamic structural equation model
Source: BMC Psychol. 2022 May 21;10:129. doi: 10.1186/s40359-022-00841-5 (PMC9123828; doi:10.1186/s40359-022-00841-5)
Supplement: Supplementary file 1 — Additional file 1. Supplementary Table 1. Sample Characteristics (n = 122). Supplementary Codes in Step1. Supplementary Codes in Step2. [file 40359_2022_841_MOESM1_ESM.docx]

**The Bidirectional Effects of Obsessive-Compulsive Symptoms and Difficulties in Emotion Regulation in Chinese Adults During the COVID-19 Pandemic: A Dynamic Structural Equation Model**

**Supplementary Materials**

**Supplementary Table 1.** Sample Characteristics (*n*=122).

| Characteristics | *n* (%) |  | Characteristics | *n* (%) |
| --- | --- | --- | --- | --- |
| Age in years |  |  | Occupation |  |
| 18-25 | 109 (89.3) |  | Student | 102(83.6) |
| 26-45 | 8 (17.6) |  | Others | 20(16.4) |
| >45 | 5 (4.1) |  | Annual Family Income |  |
| Gender |  |  | <8,000 RMB | 60(49.2) |
| Male | 53(43.4) |  | 8,000~15,000 RMB | 43(35.2) |
| Female | 69(56.6) |  | 15,000~80,000 RMB | 19(15.6) |
| Physical conditions |  |  | Highest education |  |
| Have a history of chronic illness | 11(9.0) |  | Less than high school or GED | 12(9.8) |
| Health | 111(91.0) |  | College degree or higher | 110(90.2) |
| Current residence |  |  | Other major issues |  |
| less severe epidemic areas | 56(45.9) |  | Yes | \| 40(32.8) \| \| --- \| |
| generally severe epidemic areas | 29(23.8) |  | No | 82(67.2) |
| severe epidemic areas | 12(9.8) |  |  |  |
| extremely severe epidemic areas | 25(20.5) |  |  |  |

*Note*. The classification standard of the current residence refers to the pandemic map of Dingxiangyuan ([https://portal.dxy.cn/](https://www.sohu.com/a/373867212_727571.)) from February 1 to February 15, 2020, it is divided into 4 regions: ①Less severe epidemic areas including Yunnan, Hainan, Guizhou, Shanxi, Liaoning, Tianjin, Gansu, Jilin, Inner Mongolia, Xinjiang, Ningxia, Qinghai, HongKong, Macau, Taiwan and abroad, ②Generally severe epidemic areas including Chongqing, Shandong, Sichuan, Heilongjiang, Beijing, Shanghai, Fujian, Hebei, Guangxi, Shanxi, ③Severe epidemic areas including Hunan, Anhui, Jiangxi, Jiangsu, ④Extremely severe epidemic areas including Hubei, Zhejiang, Guangdong, Henan.

**Supplementary Codes in Step1**

TITLE: step1;

DATA: FILE = step.dat;

VARIABLE: NAMES = serial day OC DER HA anxiety depress age gender;

USEVARIABLES = OC DER;

Lagged = OC(1) DER(1);

Cluster = serial;

TINTERVAL = day(1);

ANALYSIS: TYPE = TWOLEVEL RANDOM;

Estimator=bayes;

Biter=(1000);

Bseed=5682;

Processors=2;

MODEL:

%WITHIN%

P1 | OC ON OC&1;

P2 | DER ON DER&1;

P3 | DER ON OC&1;

P4 | OC ON DER&1;

OC WITH DER;

%BETWEEN%

P1 P2 P3 P4 OC DER WITH P1 P2 P3 P4 OC DER;

OUTPUT: TECH1 TECH8 STDYX;

PLOT: TYPE = PLOT1 PLOT2 PLOT3;

**Supplementary Codes in Step2**

TITLE: step2;

DATA: FILE = step.dat;

VARIABLE: NAMES = serial day OC DER HA anxiety depress age gender;

USEVARIABLES = OC DER HA anxiety depress;

Lagged = OC(1) DER(1);

Cluster = serial;

TINTERVAL = day(1);

ANALYSIS: TYPE = TWOLEVEL RANDOM;

Estimator=bayes;

Biter=(1000);

Bseed=5682;

Processors=2;

MODEL:

%WITHIN%

P1 | OC ON OC&1;

P2 | DER ON DER&1;

P3 | DER ON OC&1;

P4 | OC ON DER&1;

OC WITH DER;

%BETWEEN%

OC DER P1 P2 P3 P4 ON anxiety depress HA;

P1 P2 P3 P4 OC DER WITH P1 P2 P3 P4 OC DER;

OUTPUT: TECH1 TECH8 STDYX;

PLOT: TYPE = PLOT1 PLOT2 PLOT3;
